# Results of the feasibility phase of the managed activity graded exercise in teenagers and pre-adolescents (MAGENTA) randomised controlled trial of treatments for chronic fatigue syndrome/myalgic encephalomyelitis

**DOI:** 10.1186/s40814-019-0525-3

**Published:** 2019-12-19

**Authors:** Amberly Brigden, Lucy Beasant, Daisy Gaunt, William Hollingworth, Nicola Mills, Emma Solomon-Moore, Russell Jago, Chris Metcalfe, Kirsty Garfield, Charlotte Wray, Adam Trist, Victoria Vilenchik, Caroline Grayson, Esther Crawley

**Affiliations:** 1 Centre for Academic Child Health, Bristol Medical School, University of Bristol, 1-5 Whiteladies Road, Bristol, BS8 2BN UK; 2 Bristol Randomised Trials Collaboration, Population Health Sciences, Bristol Medical School, University of Bristol, Bristol, BS8 2PS UK; 3 Population Health Sciences, Bristol Medical School, University of Bristol, Bristol, BS8 2PS UK; 4 Department for Health, University of Bath, Claverton Down, Bath, BA2 7AY UK; 5 Centre for Exercise, Nutrition & Health Sciences, School for Policy Studies, University of Bristol, Bristol, BS8 1TZ UK; 6 Great North Children’s Hospital, Royal Victoria Infirmary, Queen Victoria Road, Newcastle upon Tyne, NE1 4LP UK

**Keywords:** Chronic fatigue syndrome, Myalgic encephalomyelitis, CFS/ME, Graded exercise therapy, Activity management, Feasibility study, Randomisation controlled trial, Qualitative research

## Abstract

**Background:**

Chronic fatigue syndrome (CFS) also known as myalgic encephalomyelitis (ME) is relatively common in young people and causes significant disability. Graded exercise therapy (GET) and activity management are recommended by the National Institute for Health and Care Excellence (NICE) despite a limited evidence-base for either treatment in paediatric CFS/ME. This paper reports on feasibility and acceptability measures from the feasibility phase of the ongoing MAGENTA randomised controlled trial (RCT) investigating GET versus activity management for young people with CFS/ME.

**Methods:**

Setting: Three specialist secondary care National Health Service (NHS) Paediatric CFS/ME services (Bath, Cambridge and Newcastle).

Participants: Young people aged 8–17 years with a diagnosis of mild to moderate CFS/ME. Young people were excluded if they were severely affected, referred to cognitive behavioural therapy (CBT) at initial assessment or unable to attend clinical sessions.

Interventions: GET and activity management delivered by physiotherapists, occupational therapists, nurses and psychologists. Families and clinicians decided the number (typically 8–12) and frequency of appointments (typically every 2–6 weeks).

Outcome Measures: Recruitment and follow-up statistics. We used integrated qualitative methodology to explore the feasibility and acceptability of the trial processes and the interventions.

**Results:**

80/161 (49.7%) of eligible young people were recruited at two sites between September 2015 and August 2016, indicating recruitment to the trial was feasible. Most recruitment (78/80; 97.5%) took place at one centre. Recruitment consultations, online consent and interventions were acceptable, with less than 10% in each arm discontinuing trial treatment. Response rate to the primary outcome (the SF36-PFS at 6 months) was 91.4%. Recruitment, treatment and data collection were not feasible at one centre. The site was withdrawn from the study.

In response to data collected, we optimised trial processes including using Skype for recruitment discussions; adapting recruiter training to improve recruitment discussions; amending the accelerometer information leaflets; shortening the resource use questionnaires; and offering interventions via Skype. These amendments have been incorporated into the full trial protocol.

**Conclusions:**

Conducting an RCT investigating GET versus activity management is feasible and acceptable for young people with CFS/ME.

**Trial registration:**

ISRCTN23962803 10.1186/ISRCTN23962803, date of registration: 03 September 2015

## Background

Paediatric chronic fatigue syndrome (CFS) also known as myalgic encephalomyelitis (ME) is relatively common, with an estimated prevalence between 1 and 2.4% in adolescence [1, 2]. It is a complex, serious and disabling condition that includes a range of symptoms such as debilitating fatigue, muscle and joint pain, flu-like symptoms, sleep difficulties and nausea [3]. It has a significant impact on young people’s lives; most (62%) of those attending specialist services attend only 2 days a week of school or less [4], over half are bed-bound at some stage [5] and affected young people give up social activities and hobbies [6].

The National Institute for Health and Care Excellence (NICE) recommends that young people with CFS/ME are offered either graded exercise therapy (GET), activity management or cognitive behavioural therapy (CBT) [7]. GET stabilises physical activity levels, before gradually increasing at a manageable rate. Activity management establishes a baseline for all activity which is then increased [7, 8]. In adolescents, this is mainly cognitive activities, such as school/homework, time on-line and social activities. CBT includes behavioural elements, but also uses cognitive approaches to support psychological needs and encourage behaviour change [9]. There is some evidence for the effectiveness of CBT in young people with CFS/ME [10–12]; however, there is limited evidence for the effectiveness of GET in this population [13]. In adults, when added to standard medical care, GET is moderately effective in reducing fatigue and improving physical function [14].

MAGENTA is a randomised controlled trial (RCT) to investigate the effectiveness and cost-effectiveness of GET versus activity management in outpatient treatment of paediatric CFS/ME. The study is evaluating complex interventions [15]; the interventions have several interacting components, and there is a degree of flexibility in how the interventions can be delivered. MAGENTA includes process evaluation [15], including mediation analysis and testing trial processes such as a novel method of online consent. Prior to the full-scale RCT, we carried out a feasibility study [15]. This was to determine the feasibility of trial processes and whether the trial and interventions were acceptable to young people with CFS/ME. Findings from feasibility studies can be used to improve processes for the full trial, for example improving recruitment consultations, refining outcome measures and guiding the delivery of interventions [16–18]. If there were no substantial changes to trial methodology or the delivery of interventions, we planned to use data from the feasibility phase in an adequately powered RCT. In this paper, we report the results from the feasibility phase of the trial.

## Aims and objectives

To ascertain the feasibility and acceptability of conducting an RCT to investigate the effectiveness and cost-effectiveness of GET compared to activity management for paediatric CFS/ME with the aim of moving seamlessly to a full RCT. Specific objectives were to:

1. Assess the number of young people who were: eligible, approached, recruited and retained in the first six months of the study.
2. Identify barriers and facilitators to recruitment.
3. Explore issues of retention and understand why people drop out of the study.
4. Assess the acceptability of intervention.
5. Evaluate the fidelity of intervention delivery.
6. Assess the feasibility and acceptability of using accelerometers.

## Methods

### Design

We conducted a feasibility study with integrated qualitative methods. So long as the interventions and study processes were not significantly changed on proceeding to the full trial, this initial stage would be considered as an internal pilot and the outcome data collected included in the main trial analysis. Full details of the methods can be found in the published protocol [19] and are summarised below.

### Setting

Recruitment to the feasibility study occurred between September 2015 and August 2016 at three Specialist Paediatric CFS/ME National Health Service (NHS) services: Bath, Newcastle and Cambridge. Combined, these services provide assessment and treatment for more than 380 young people each year.

### Participants

Young people were screened for eligibility at their initial clinical assessment carried out by a CFS/ME clinical specialist (including paediatricians and psychologists). Young people were eligible if they:

- Had a diagnosis of mild to moderate CFS/ME. [7]
- Were aged between 8–17 years.

And excluded if they:

- Were severely affected (unable to do activity for themselves, only able to carry out minimal daily tasks, or had severe cognitive difficulties and depend on a wheelchair for mobility. [7])
- Referred to CBT at their first clinical assessment.
- Were unable to attend clinical sessions.

### Procedure

**Fig. 1:**
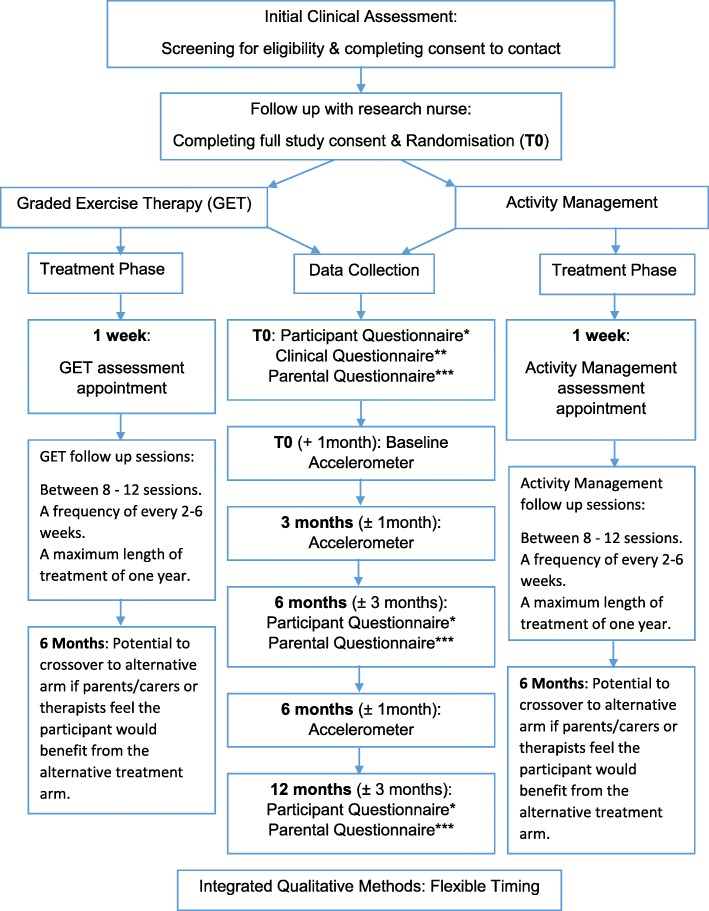
Study flow diagram detailing participant flow through clinical and research processes. From the MAGENTA protocol [19]

Assessing clinicians identified potential participants, provided an overview of the study and gained assent/consent to contact from those families interested in learning more about the study. Full recruitment discussions, typically carried out by a specialist nurse, were conducted face-to-face or via telephone/Skype. At the outset, the recruiter confirmed that the families were happy for the discussion to take place and happy for it to be audio-recorded. They continued to discuss information about the trial: study design, interventions, participant burden and the potential benefits and risks. From the outset, our aim was to continue seamlessly into a full trial if this RCT was shown to be both feasible and acceptable. Participants were therefore informed that their outcome data would be used in the full trial if the RCT was shown to be feasible. Families wishing to consent to the study could do so by completing online consent forms via the REDCap (Research Electronic Data Capture) hosted at the University of Bristol [20]. Young people under the age of 16 provided assent and those over 16 provided consent. We also obtained consent from carers/parents. Paper consent forms were used for face-to-face recruitment consultations. Consenting participants were then randomised, using the automated web service operated by the Bristol Randomised Trials Collaboration. Allocation (1:1) used minimisation to facilitate balance by age and gender, and retained a random component to prevent accurate prediction of allocation. Because of the nature of the intervention, it was not practical to keep either the family or the clinical service blind to treatment allocation. Participants were informed of their allocation either at the end of the recruitment consultation, or at a later date by phone. Figure 1 describes the trial and treatment processes.

### Sample size

We calculated that a sample size of approximately 100 participants would provide sufficient information to inform a full trial. Recruitment of 100 participants from 430 young people assessed for eligibility would give a 95% confidence interval of the order of 20–28% for estimating a true recruitment rate (rate of those assessed found to be eligible and subsequently consented) of 24%. Twenty-four percent is reasonable, based on previous RCTs exploring treatment for paediatric CFS/ME [21].

### Interventions

In both arms, clinicians could provide routine advice about sleep, medication use and symptom control. Families and clinicians decided upon the number of follow-up sessions (typically between 8 and 12) and the frequency of appointments (typically every 2–6 weeks). The interventions were delivered in secondary care outpatient clinics, delivered face-face in the hospital setting or via Skype (see section “The acceptability (satisfaction and adherence) of intervention”).

#### Graded exercise therapy

Trained GET therapists (physiotherapists, occupational therapists, nurses or psychologists) initially assessed the young person’s physical activity and collaboratively recommended a tailored treatment plan. This started with identifying a “baseline” of physical activity. The baseline is the average level of physical activity that a young person does. It is normally about half of what they can do on a good day. Establishing a baseline, means the young person will do the same level of physical activity each day, avoiding “payback” or an increase in symptoms which usually occurs after they have done too much. It therefore avoids the boom-bust pattern of exercise (doing too much, followed by an increase in symptoms and doing not very much) that is typical of CFS/ME. In addition, therapists calculated the young person’s “maximum” heart rate (220 minus their age). At the start of treatment, young people were advised to try and ensure physical activity levels were low enough so heart rate did not increase to more than 40–50% of this maximum heart rate. Clinicians taught young people how to manually measure their heart rate. Younger children and those unable to manually take their heart rate, were offered a Fitbit Charge HR [22] to assist this (subject to availability).

Once the baseline level of activity was achieved and maintained, the young person was supported to gradually increase physical activity, increasing activity levels by 10–20% a week [7].

#### Activity management

Activity management was delivered by specialist CFS/ME clinicians (occupational therapists, physiotherapists, nurses and psychologists). The clinician assessed the participant’s current levels of activity, including cognitive activities (school work, reading, socialising and screen time (phone, laptop, TV, games)), emotional activities (for example, having an argument) and physical activities. The clinician and participant then agreed a “baseline” of activity: a daily sustainable level of activity, typically the average daily amount of activity that the young person reported at assessment. Young people were taught how to record the total number of minutes spent each day doing different levels of activity (high-energy and low-energy) using either paper diaries or the “ActiveME” digital App. When participants achieved a “baseline” of all activity (cognitive/physical/emotional), they were supported to gradually increase activity by 10–20% each week.

### Data collection

#### Screening, eligibility, consent and randomisation

We recorded the number of potentially eligible participants attending the clinic, the number assessed for eligibility, the number of eligible patients who consented (and the reasons why families declined) and the number who were randomised. We also recorded the number of participants who discontinued treatment and the number who completed outcome measures. These statistics were presented in Consolidated Standards of Reporting Trials (CONSORT) flow charts.

#### Patient-reported outcome measures

At baseline, six and 12 months, the following data were collected from participants via self-report questionnaires: physical function (SF36-PFS) [23]; fatigue (Chalder scale) [24]; educational attendance (self-report school or home tuition); mood (Hospital Anxiety and Depression Scale) [25]; (Spence Children’s Anxiety Scale) [26]; pain (visual analogue scale) [27]; (Clinical Global Impression Scale) [14]; general health-related quality of life (EQ-5D-Y) [28]. The anticipated primary outcome for the main trial comparison was the SF36-PFS at six months.

At baseline, six and 12 months, parents/carers were asked to complete questions about their child’s healthcare resource use, and the Work Productivity and Activity Impairment Questionnaire: General Health (WPAI:GH) to capture the effect of their child’s health problems on their ability to work and perform regular activities [29].

Baseline participant self-report questionnaires were collected on paper forms prior to randomisation. All other self-report questionnaires were completed remotely via REDCap, a secure system used by many institutions for large multicentre studies. Participants submitted their questionnaire data directly to the REDcap system. If questionnaires were not completed, a researcher contacted the family and asked to complete the primary outcome data over the telephone.

#### Accelerometer

Participants in both trial arms were asked to wear an accelerometer (Actigraph GT3X+, Actigraph LLC Florida) to measure physical activity for seven days within 1 month of randomisation and at three- and six-month follow-up. Accelerometers are small, match box-sized devices that measure physical activity. The device is attached to a waist band and sits on the hip. The accelerometer data were processed to identify the number of participants who supplied valid data. Data were deemed valid based on the procedures used in the International Children’s Accelerometer Database (ICAD) if participants wore the accelerometer for at least two weekdays and at least one weekend day out of the seven, for at least 500 min per day [30].

#### Quantitative data analysis

Baseline continuous data were summarised by median and interquartile range and categorical data by counts and percentages. No data analysis of outcome measures was performed at the feasibility stage because the data were to be retained for use in the main trial.

### Integrated qualitative methodology

#### Recruitment consultations

Recruitment consultations were routinely audio-recorded to explore information provision, and the acceptability of trial methodology (e.g. randomisation). During the feasibility phase, three recruiters for the three sites received one-to-one recruitment training (1.5 to 4.5 h each) from a member of the research team (LB). Training was based on the communication strategies shown to be effective in trials of adults in terms of engaging with treatment preferences and conveying equipoise [17, 31–33]. Recruitment consultations that highlighted good practice or potential barriers to recruitment were transcribed and discussed with recruiters at each training session. A “Tips for recruitment and informed consent” document was developed and given to each recruiter as a guide for good practice.

#### Participant interviews

Semi-structured interviews were conducted with participants and parents who had consented to the trial to understand their views and experiences of trial processes. This included acceptability of patient information, treatment interventions and use of accelerometers/heart rate monitors. Participants were offered a choice of interview location: at home, in the hospital, via skype or by telephone. A checklist of topics was developed from a previous trial carried out with young people with CFS/ME [34]. This was used to guide discussion, but those participating were encouraged to raise issues they felt to be important. Interviews lasted between 15 and 60 min. They were audio-recorded with consent, and transcribed verbatim.

#### Qualitative data analysis

Qualitative data analysis was an on-going, and iterative process that started soon after data collection began, using techniques of constant comparison to inform further sampling and data collection [35]. Recruitment consultations were purposively selected for analysis on a month by month basis, representing a mix of families who accepted and declined randomisation. Attention was paid to consultations in which families declined the trial, to understand the views of families who opted for treatment outside the trial. As the trial progressed, consultations that highlighted issues of trial acceptability (such as crossover and study withdrawal) were analysed for content and presentation of information. Thematic analysis [36] was used to identify common or divergent themes, particularly focusing on the impact of information delivered by recruiters, on patients and parents. Individuals exhibiting contrasting views (negative cases) were studied in detail to understand reasons underlying such differences [37]. Interview transcripts were imported into NVivo 10 and analysed thematically in parallel with the corresponding recruitment consultation, to explore the acceptability of trial methodology and determine feasibility of a full trial.

### Fidelity checks

Intervention sessions were routinely audio-recorded, with consent. We transcribed these recordings, removed patient and clinician identifiable data and removed the words “Graded Exercise Therapy” and “activity management” to blind those rating the transcripts to treatment allocation. Using the protocol, we created a checklist of GET elements and activity management elements. Clinicians were asked to review the blinded transcript against this checklist and then record whether they thought it was a GET or activity management session.

### The Trial Steering Committee and the Data Safety Monitoring Committee

The Trial Steering Committee (TSC) (who met three times during the feasibility phase) were responsible for advising on trial methodology, reviewing the progress of the study against the Stop-Go criteria and advising whether the feasibility study should proceed to the full trial. The Data Safety Monitoring Committee (DSMC) met once during the feasibility phase to review recruitment, retention, withdrawal rates and safety outcomes. Neither the Trial Management Group (TMG), TSC nor the DSMC analysed any patient-reported outcome data by trial arm during the feasibility phase of the study. The DSMC analyses of safety outcomes were conducted by the study statistician, who took no part in the decision to use the feasibility data in the full trial.

### Patient involvement

We consulted with the University of Bristol CFS/ME Young Persons Advisory Group (YPAG) prior to the study to gain feedback on aspects of trial design (telephone recruitment) and the study documents.

## Results

Between September 2015 and August 2016, 287 young people were assessed across the three sites (for site details see below). Of these, 161 were eligible for the study and 80 (49.7% of those eligible) were recruited. Table 1 provides the baseline characteristics of those recruited into MAGENTA. Seventy percent of the participants were female, with a median age of 15 years, median illness duration of 15 months and median school attendance of 3.0 school days per week. The baseline measures were nearly all complete. Two participants reported that school attendance was not applicable, one of whom was receiving home tuition and one was not. One participant missed all items of the Chalder Fatigue scale. Fourteen participants were missing all items of the HADs anxiety and HADs depression subscales.

**Table 1:** Baseline characteristics of participants

Outcome	Median (25 percentile, 75 percentile) or *n* (%)	*n*
Age in years	15.0 (12.5, 16.0)	80
Female	56 (70.0%)	80
Ethnicity; White or British	76 (95.0%)	80
Months since onset of symptoms	15.0 (10.0, 30.0)	80
School attendance, days per week	3.0 (1.0, 4.0)	77
Home tuition; yes	10 (12.5%)	80
SF36 Physical Function	55.0 (37.5, 72.5)	80
Chalder Fatigue	25.0 (22.0, 28.0)	79
Spence Children’s Anxiety Scale	32.0 (19.5, 47.0)	80
HADS Anxiety	8.0 (6.0, 13.0)	66
HADS Depression	8.0 (4.0, 11.0)	66

Interviews were conducted with 27 families from Centre One. Twenty-six interviews were conducted with parents (24 mothers, one father and one joint interview with both parents) and 26 with young people participating in the trial (10 male and 16 female). Three families cancelled interviews because they were unavailable, one participant declined to be interviewed on the day and one parent was unavailable for interview. Two participants had discontinued their allocated treatment at the time of interview (both assigned activity management). Participants were asked if they were willing to be interviewed alone but 21 chose to be interviewed with their parent present. Ten families chose face-to-face interviews (nine in their home and one at their local hospital); 14 were interviewed over the phone and three via Skype. Families were interviewed at varying time point in the treatment period, ranging from 3 to 35 weeks post randomisation.

### Eligibility and recruitment

The feasibility of recruitment was assessed in all three sites.

#### Centre One

**Fig. 2:**
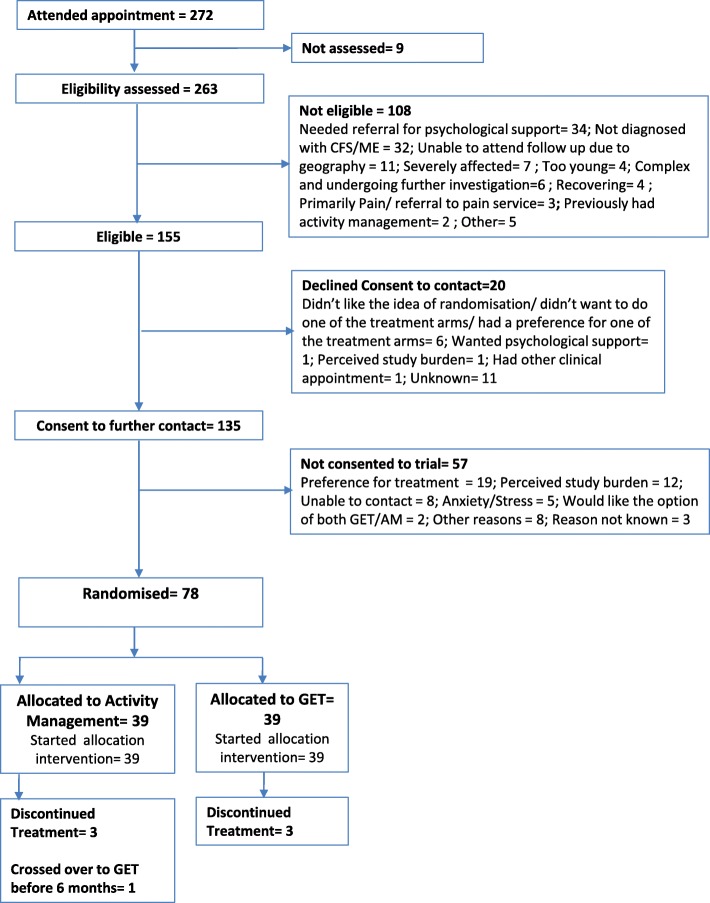
CONSORT diagram for Centre One

Between 1 September 2015 and 31 August 2016, 272 young people attended clinic appointments of which 155 (57.0%) were assessed as being eligible. The main reasons for exclusions were referral for psychological support at assessment (34), not diagnosed as having CFS/ME (32) and being unable to attend follow up (11). Of the 155 eligible, 78 (50.3%) consented and were randomised to the study. The main reasons families declined participation after discussion with the researcher were preference for a specific treatment (19) and perceived study burden (12). The number of participants randomised was therefore 78 out of the 263 assessed for the study (30%, 95% confidence interval 24%, 36%). Figure 2 describes the flow of young people screened and recruited at Centre One

#### Centre Two

Centre Two was open to recruitment between January 2016 and 31 August 2016. Nine young people were assessed within the service, three of whom were ineligible (two were unable to attend follow-up and one had severe CFS/ME). Of the six eligible patients, three declined before consent to contact and one declined at the recruitment discussion. All three patients cited the distance of travel to the service as the reason for declining. Two participants consented to the trial. See Additional file 1 for the CONSORT diagram.

#### Centre Three

Centre Three started recruiting in January 2016. Six participants were assessed within the service. Three were not eligible for MAGENTA; reasons for exclusion were referral to psychological services, previously seen in the service and too old. Three participants were recruited. Clinicians were unable to deliver treatment, deliver recruitment calls or collect baseline data according to the protocol. Therefore, the three patients were withdrawn from the trial but continued to receive specialist medical care. See Additional file 2 for the CONSORT diagram.

#### Provision and acceptability of patient information and recruitment process

Participants provided positive feedback about being involved in the research:

“it’s quite fun…you’re also helping other people with CFS in the long run” (young person, ID108, activity management)

Participants and their families felt the oral recruitment consultation was acceptable, and felt they provided the “right level of information” in a “straightforward” and “well explained” manner. Participants indicated that the recruitment consultation enabled them to ask further questions and “clarify” what participation entailed.

The participants who read the patient information leaflets found them acceptable. Some participants reported that they could not remember or did not read the information sheet and relied on the fact that their parent(s) had read it:

“I didn’t read it too much but mum did and she looked quite happy with it” (young person, ID29, activity management)

The majority of parents and participants found the online consent system (REDCap) acceptable and easy to use. Most preferred this method of consent over paper consent forms, but some found the online system more difficult to use on a tablet or smartphone:

“I tried to do it on my phone but yeah, it was just far too small on my phone … it was quite difficult [on a tablet] because obviously ticking, when you’re trying to like tap the bits in the box, sometimes you press something and it’s, yeah…it disappeared at one point” (mother, ID65, activity management)

### Retention

In the first 12 months of the study, 35 participants were due to complete their six-month follow-up questionnaires. 91.4% (32) of participants completed their 6-month primary outcome measures. Three participants withdrew from research follow-up, for unknown reasons.

At six months, of the 35 healthcare resource use and work productivity questionnaires due to be completed, 15 (42.9%) were returned. To maximise response rate, we reduced and simplified these questionnaires. We removed the adapted healthcare resource use questions and the socioeconomic questions from the baseline questionnaire. We also reduced the level of detail required from participants about their medication use (removing dosage and route of administration), and changing the question from a free text response, to a multiple-choice response (with the option of “other” and free text).

### The acceptability (satisfaction and adherence) of intervention

All participants started the treatment that they were allocated. Three out of the 40 participants in the activity management arm (7.5%) and three out of the 40 participants in the GET arm (7.5%) discontinued treatment early (defined as discontinuing treatment within six months of randomisation). Participants could indicate more than one reason for discontinuing treatment. Reasons given were the following: preference for the other arm (4), not recovering in the allocated arm (2), deteriorating in the allocated arm (1), not wanting further clinical treatment with the service (1).

One participant in the activity management arm crossed over to the GET arm after six months of treatment because of a preference for this treatment. This family was interviewed shortly after joining the trial; the participant was happy to continue to participate at this time, but all three family members (participant, mum and dad) discussed a preference for the non-allocated treatment arm. This was largely due to a feeling that they had already tried similar techniques:

“I kind of wanted to be in the other, other side of the trial […] because I’d already tried this side and it hadn’t worked before, so I was sort of wondering if maybe like the other side would work. But, I’m happy to try this side [activity management] and see if it works better, now I’m older kind of thing”(young person, ID35, activity management).

The mean (SD) number of appointments attended at Centre One was similar between groups (activity management 9.7 (4.7), *n* = 37 and GET 9.6 (4.5), *n* = 39) (note that data on number of appointments attended was missing for two participants). 

Participants and parents in both arms commented on finding core aspects of the specialist CFS/ME care package beneficial, (such as sleep hygiene, referral to a psychologist and information about diet) and the importance of a positive and trusting relationship with their clinician:

“[clinician] goes through a lot more than just the exercise, [GET] like I have like issues with my diet and stuff, and she looks at that. Does the whole sleep… like she referred me to a psychologist so there’s actually a lot more which I wasn’t expecting for it to cover. Which is really good, like that’s helped quite a lot” (young person, ID20, GET).

“It’s focussed a lot on sleep. […] she’s definitely moved my sleep time and that’s been the most helpful part*”* (young person, ID9, activity management).

Qualitative feedback supports the acceptability overall of both intervention arms. Although some families expressed initial anxiety about the prospect of GET, most were satisfied that it would be manageable, “flexible” and tailored to the individual:

“It’s been really flexible to meet his needs so erm, the… the exercise initially it was increased because [name] could cope with that at the time and it’s decreased with, you know, [name] needs changed… cause he was coping with other things as well, and so erm, the exercise has decreased to allow for that for the moment and I’m very confident that *when* we go back [clinician] will listen to everything he says and… and you know, change it accordingly and appropriately really” (mother, ID43, GET).

Some participants reported that the GET programme required them to initially reduce the amount of activity they were doing, and they found it hard to *“restrict”* their physical activity. They wanted to do more or did not realise how much physical activity they were already doing until they started to monitor it via GET:

Interviewer: Have you been given … a level of activity and exercise that you’re supposed to do every day?

Young person: I don’t like the level.

Mother: No. She wasn’t happy.

Young person: No, it’s not good.

Mother: Ballet is one of … that’s her number one thing that she loves more than anything in life and she’s had to cut back a lot on her ballet, which I didn’t know they’d say that she had to do and she’s really not very happy about it….

(mother and young person ID5, GET).

Participants were encouraged to build up slowly to avoid a “boom and bust” pattern of physical activity. Most reported feeling less tired generally when they had established a manageable and consistent amount of activity on a daily basis:

“It’s [GET] been helpful. I can certainly walk further now without being as tired. It’s stopped me being quite as lethargic, getting out every day, definitely, and yeah, my fitness has improved” (young person, ID25, GET).

Those in the activity management arm also found the approach positive and helpful on the whole when managing CFS/ME symptoms. They also highlighted that “limiting” high level “red” activity could be challenging and frustrating, particularly in the run up to school exams. Recording cognitive activity levels on activity management sheets or the “ActiveME” App was seen as onerous for some, although parents and participants noted that overall activity management had a beneficial effect on their CFS/ME:

“She’s now reading books, she’s now able to cope with watching, you know, something on television that she hasn’t seen before. She’s now coping with maths, – and [name] is much happier I think” (mother, ID51, activity management).

During the feasibility phase, Centre One started to offer appointments via Skype so that young people did not have to travel to appointments. Clinicians felt that this could also be beneficial for participants within the MAGENTA study. As such, the MAGENTA protocol was amended to allow the use of Skype, and this amendment was approved by ethics.

#### Acceptability of the Fitbit [heart monitor, GET only]

The original intention was to provide every participant in the GET arm of the study with a Fitbit to measure their heart rate, but this did not prove feasible because fewer participants than anticipated returned them. Therefore, the protocol was amended so that clinicians taught young people to manually measure their heart rate. Younger children and those who were unable to measure their heart rate were provided with a Fitbit (subject to availability). This was incorporated into the full trial protocol.

Those randomised to GET reported that they enjoyed using the Fitbit, often finding other functionality such as sleep or steps monitoring useful in addition to heart rate monitoring. Some participants were keen to be randomised to GET so they could use a Fitbit; “He just wanted the FitBit, that was all he wanted” (young person, ID23,GET). One family in the activity management arm bought a Fitbit for their child shortly after joining the trial. However, one participant found the Fitbit too uncomfortable to wear at night; “Well it’s hard to wear during the night because I’m on my hand a lot, and then it like hits my bone” (young person, ID16, GET)*.*

### Fidelity of interventions

Twelve treatment sessions were sampled from Centre One, selecting two sessions from each clinician at random. One session was sampled from Centre Two. Of the 13 sessions rated by clinicians blinded to intervention, all were correctly identified as the allocated treatment.

### The feasibility and acceptability of accelerometers

Acceptability and feasibility were determined by the amount of valid data supplied by participants [30].

Of those accelerometers returned, we looked at the number of participants who provided valid data at baseline, three and six months.

Table 2 shows that at baseline, 39 participants (66.1%) provided valid data. Of the 20 participants (33.9%) who did not provide valid data, 5 (8.5%) did not wear the accelerometer at all. Fifty-two participants returned an accelerometer at three months, of which 33 (63.5%) provided valid data, while three (5.8%) did not wear the accelerometer at all. At six months, 45 participants returned an accelerometer; 19 participants (42.2%) provided valid data and seven (15.6%) did not wear the accelerometer at all.

**Table 2:** Accelerometer valid wear time and non valid wear time (based on at least 500 min per day of data for at least 2 weekdays and a weekend day)

Time-point	Valid use, *n* (%)	Non-valid use with some data available, *n* (%)	Non-valid use with no data available, *n* (%)	Total
Baseline	39 (66.1)	15 (25.4)	5 (8.5)	59
3 months	33 (63.5)	16 (30.8)	3 (5.8)	52
6 months	19 (42.2)	19 (42.2)	7 (15.6)	45

Participants had mixed views on the acceptability of the accelerometer. Some participants didn’t mind wearing it and felt: “No one can see it, um it was very discreet it went under my clothes” (young person, ID9, activity management)*.* Others found it “a bit un-comfy” (young person, ID5, GET) or “itchy” young person, ID72, activity management) and would have liked it to be more discreet; “I struggled a bit to find baggy enough clothes to wear with it” (young person, ID25, GET). Some participants had not told friends about their CFS/ME and the accelerometer raised unwanted questions; “I don’t like people asking questions all the time, about that sort of thing” (young person, ID25, GET) and had the potential to make their condition more visible to others; “So I did get a few odd looks ‘What are you wearing?’” (young person, ID20, GET)*.* Some participants forgot to put the belt back on in the morning, after swimming or a shower; “I often forgot to wear it a few times” (young person, ID29, activity management)*.* Some participants reported that they did not wear the accelerometer if they felt it was not a “typical” week (young person, ID129, activity management). To address these issues, we amended the “Accelerometer information sheet” in July 2016 to encourage participants to wear the accelerometer whether it was perceived as a typical week or not.

### Ethical amendments

There were three substantial ethical amendments; full details can be found in Table 5 in Appendix 1 and a summary is provided in Table 3.

**Table 3:** Summary of amendments

Amendment number, date of ethics committee approval	Summary of amendment (see Appendix 1 for full details of all substantial amendments)
1. 29 January 2016	Amendments were made to the consent to study forms for parents of participants age 16–17: Participants aged 16–17 did not need parents to consent on their behalf; however, parents were being asked to complete parental questionnaires and needed to provide consent to this research procedure. A consent form for parents/carers of young people aged 16–17 years was added. Amendments were made to the consent to study forms: We intended to use data collected during the feasibility study for the full-scale trial. This was stated in the protocol and the participant information sheets. The consent to study form was updated to reflect this. Amendments were made to the protocol: We amended the protocol to allow qualitative interviews via Skype. Amendment were made to the consent to contact, and consent to discussion forms: Changed wording and field added.
2. 31 March 2016	Amendments were made to the protocol: Amendments were made to protocol in response to reviewers’ comments upon the publication of the MAGENTA feasibility protocol. This included changes to the background information, aims and objective, the methods, adding stop-go criteria and safety outcomes.
3. 27 July 2016	Amendments were made to the health economics forms: We amended the forms due to poor response rate. We developed a second version of the baseline, 6-month and 12-month questionnaires. The new forms continued to capture our primary outcomes. Amendments were made to the protocol to document the closing down the Cambridge Site: We were unable to recruit from the Cambridge site and documented the sites closure in the protocol. Amendments were made to the protocol: We changed wording in the protocol to provide further detail and clarification in the background and methods sections.

### Feasibility criteria

The stop-go criteria (as reported in our protocol) are shown in Table 4, with an assessment of whether the criteria were met.

**Table 4:** Assessment of feasibility stop-go criteria

Stop-go criteria	Assessment	Stop-go-amend
Stop criteria: Less than 70 children and adolescents have been recruited (∼ 70% of the target) and if the qualitative data collected suggest that recruitment cannot be improved any further.	80 participants were recruited to the study.	Go
Stop criteria: The 6-month follow-up is < 80% and if the qualitative data suggest that follow-up rates cannot be improved any further.	91.% (32) of participants completed their 6-month primary outcome measures	Go
Stop criteria: Data suggest the interventions are not acceptable to children and/or their parents.	All participants started the treatment that they were allocated. Three out of the 40 participants in the activity management arm (7.5%) and three out of the 40 participants in the GET arm (7.5%) discontinued treatment early (defined as discontinuing treatment within 6 months of randomisation). Qualitative feedback supports the acceptability overall of both intervention arms.	Go, Amendments: 1. Treatment sessions could be carried out via Skype. 2. Fitbits only offered routinely to younger children and those unable to manually measure their heart rate (subject to availability).
Stop criteria: If the Data and Safety Monitoring Committee (DSMC) and the Trial Steering Committee (TSC) recommend the trial is stopped for safety reasons.	The TSC, DSMC and TMG concluded that the trial methodology and interventions were acceptable and feasible, that no significant changes needed to be made to either the interventions or outcomes and that recruitment should continue seamlessly to the full trial. As participants have consented to the use of data in the full trial, and neither the interventions nor outcomes had changed; the decision was made (with the support of the DSMC, TSC and TMG) to use the outcome data collected during the feasibility RCT in the full trial.	Go

## Discussion

This study has shown that it is feasible to conduct an RCT investigating the effectiveness and cost-effectiveness of GET compared to activity management. Fifty percent of those who were eligible to take part consented, and families accepted the novel process of online consent, which enabled participants to be recruited at home, reducing study burden. Recruiters were trained in recruitment practice following scrutiny of audio-recorded recruitment discussions to ensure that families had sufficient and balanced information to make a fully informed decision about participation. Response rate to the primary outcome was high, in contrast to collecting accelerometer data and healthcare resource use and work productivity data. A proportion of the accelerometers (41.7% across the three time points) did not contain valid data, and some participants did not like the feel or look of the device, and the fact that this made their condition visible to peers. However, as accelerometer and healthcare resource use data were not our primary outcome, this does not affect the feasibility of conducting the full RCT. The qualitative data showed that some families had negative perceptions of GET prior to joining the study. However, those that received GET reported that it was acceptable, describing it as flexible, tailored and manageable. Equally, participants found activity management acceptable. In both arms, some participants disliked the initial restrictions on activity. All participants started their allocated treatment and less than 10% in each arm discontinued treatment, which is generally considered acceptable [38].

This is the first study to conduct a trial comparing GET and activity management for paediatric CFS/ME, utilising RCT methodology with integrated qualitative methodology. Qualitative data allowed us to gather rich data about the trial process and intervention and enabled us to improve the feasibility and acceptability and to inform the design of an adequately 0powered trial. Qualitative research methodology embedded in adult RCTs has demonstrated that exploring reasons for trial refusal and addressing patient concerns about terms that may be “misunderstood” (such as “Graded Exercise Therapy”) can improve the consent process and in turn recruitment [16, 32, 39]. Elements of The QuinteT Recruitment Intervention (QRI) [32] were used during the feasibility stage of the MAGENTA trial, e.g. regular analysis of screening, recruitment and retention figures, audio-recording recruitment consultations and conducting interviews with families and members of clinical teams. The QRI approach was drawn upon to explore trial processes, promote good communication practices and understand preferences for treatment impacting recruitment and retention. To our knowledge, this was the first time elements of the QRI were used during a feasibility trial recruiting young people.

Whilst this is a relatively small study, it was a feasibility study, and a full scale adequately powered study is underway. It became clear that it was not feasible to run the study at one site (the site was subsequently withdrawn from the study). The majority of patients were recruited from one centre. This centre covers a wide geographical region, provides treatment through 10 different NHS trusts and is a national referral service. As such, it provides assessment and treatment for many more patients than the two other centres involved in recruiting. Therefore, recruitment to a multi-centre RCT, which would increase the generalisability of findings, will be challenging.

Recruitment rates for MAGENTA were higher than our previous trial that evaluated other interventions for young people with CFS/ME, where fewer than 30% of eligible young people were randomised [21], suggesting potential participants found the trial and the interventions acceptable. Wearing an accelerometer made some participants feel that their CFS/ME was more visible to friends at school, which may have resulted in them feeling reluctant to wear the device. This issue with acceptability of accelerometers differs from previous school-based studies, which have asked all pupils in a class to wear the device [40, 41]. The low rate of adverse events, which was similar in each arm, is consistent with previous studies investigating exercise treatments [42].

The TSC, DSMC and TMG concluded that the trial methodology and interventions were acceptable and feasible, that no significant changes needed to be made to either the interventions or outcomes and that recruitment should continue seamlessly to the full trial. As participants have consented to the use of data in the full trial, and neither the interventions nor outcomes had changed, the decision was made (with the support of the DSMC, TSC and TMG) to use the outcome data collected during the feasibility RCT in the full trial. The aims of the full trial were to assess the effectiveness and cost-effectiveness of GET versus AM.

### Recommendations for a full study

- Results from this feasibility study show that this RCT is feasible and can proceed to full trial. We recommend the following changes, with the aim that these changes will improve the recruitment consultation, increase the response rates to certain outcome measures (accelerometers and health economics forms) and reduce the burden of intervention sessions. The audio recordings of recruitment consultations provided opportunities for training recruiting staff. Training aimed to address equipoise and improved the provision of balanced study information and informed consent.
- In response to qualitative feedback, we offered recruitment consultations via Skype as well as telephone to improve communication and the participant’s experience.
- To address the low response rate to the resource use and work productivity questionnaires, we shortened the questionnaires with the aim of making them more acceptable to participants.
- Feasibility and acceptability of accelerometers were limited. We amended the participant information leaflet following insights from the qualitative research to minimise issues. Future studies investigating paediatric CFS/ME may consider alternative methods for collecting activity data.
- To reduce burden on participants, we amended the study protocol so that treatment sessions in both arms could be carried out via Skype.
- It was not feasible to provide a Fitbit to every GET participant. Therefore, the protocol was amended so that clinicians taught young people to manually measure their heart rate. Younger children and those who were unable to measure their heart rate were provided with a Fitbit (subject to availability).
- Outcome data collected in the feasibility phase will be used in the full trial analysis (internal pilot, as outlined in the protocol).

## Conclusions

Conducting an RCT investigating GET versus activity management using novel techniques for recruitment is feasible and acceptable for young people with CFS/ME. As GET is recommended by NICE, an adequately powered trial is necessary to test the effectiveness and cost-effectiveness of GET for young people with CFS/ME.

From the MAGENTA protocol [19]

## Supplementary information

**Additional file 1:.** CONSORT: Centre Two.

**Additional file 2:.** CONSORT: Centre Three.

## Data Availability

The authors had access to all the data. The corresponding author had full access to all the data in the study and had final responsibility for the decision to submit for publication. Data will be made available after the full trial has been published. Given the nature of this dataset, access will be controlled. Requests will be referred to the University of Bristol Data Access Committee for approval before data can be released under an appropriate data access agreement.

## Appendix 1

**Table 5:** Summary of amendments

Amendment number	Date of ethics committee approval	Summary of amendment
1.	29/01/16	1. Consent to contact forms MAGENTA 8-15 assent to contact 11082015 d0.6 MAGENTA 16-17 consent to contact 11082015 d0.6 * MAGENTA parent carer consent to contact 11082015 d0.6 * The above 3 documents currently have a box for “research ID” this needs to be changed to “consent ID”. We are also changing the wording from “research nurse” to “research team”. In addition, the 2 consent documents marked with * need a section where participants/ parents can document the participants date of birth. We would therefore like to change the above three documents to: MAGENTA 8-15 assent to contact 18112015 d0.7 MAGENTA 16-17 consent to contact 18112015 d0.7 MAGENTA parent carer consent to contact 18112015 d0.7 2. Consent to study forms for parents of participants age 16-17 Participants aged 16-17 do not need parents to consent on their behalf; however, parents are being asked to complete parental questionnaires and do need to provide consent to this research procedure. We have therefore designed the consent form: MAGENTA parent carer consent to study (16-17) 18112015 d0.1 3. Consent to study forms MAGENTA 16-17 consent to study 18112015 d0.5 MAGENTA parent carer consent to study 18112015 d0.5 We will use data collected during the feasibility study for the full scale trial. We need participants to give their consent and will now include the wording “I agree that data collected about me can be used in the full trial”. We are also changing the wording from “research nurse” to “research team”. See new forms below: MAGENTA 16-17 consent to study 23102015 d0.6 MAGENTA parent carer consent to study 23102015 d0.5 This info has also been included in: MAGENTA parent carer consent to study (16-17) 18112015 d0.1 4. Consent to discussion MAGENTA 8-15 assent to record discussion 18112015 d0.7 MAGENTA 16-17 consent to record discussion 18112015 d0.7 We are changing the wording from “research nurse” to “researcher”. Clearer instruction on how to complete the form have been provided with the line “Please answer the following questions by circling ‘yes’ if you agree”. The line “I confirm that I would like to hear more information about this study” had been removed because it is unnecessary. “I understand that I can switch off the recorder or stop the discussion without having to give a reason”. The wording of this line has been amended to make it more child-friendly. The parental consent box has been removed from the 16-17 consent form, because it is unnecessary. 5. Protocol We may carry out qualitative interviews via Skype. We have therefore added the following sentence to the protocol: “Interviews will take place in clinic, in the participant’s home or via Skype.” The wording “special clinical psychologist” has been changed to “specialist psychologist” in line with the services practice.
2.	31/03/16	The following amendments were made to the protocol: 1. Additional background information has been included. This provides further clarification on the current evidence based and better defines the treatments. The protocol has been changed from: “NICE recommends that children with CFS/ME are offered either CBT, Graded Exercise Therapy (GET) or activity management effective in adults with CFS/ME but there is no evidence for the effectiveness or cost-effectiveness for paediatric CFS/ME. There is also limited evidence on the acceptability of GET for children with CFS/ME or on the best method for delivering GET in terms of intensity (frequency of sessions) and length of treatment (number of sessions and length of time for follow up).” to: “NICE recommends that children with CFS/ME are offered either Cognitive Behavioural Therapy (CBT), Graded Exercise Therapy (GET) or Activity Managemen t[5]. GET stabilises physical activity levels, before gradually increasing at a manageable rate. Activity management establishes a baseline for all activity (mainly cognitive, such as school and homework, in children and adolescents) which is then increased [5, 7]. There is good evidence for the effectiveness of CBT in children with CFS/ME [8–10]; however, there is no evidence for the effectiveness of GET in children although GET is moderately effective in adults [11]. There is also limited evidence on the acceptability of GET for children with CFS/ME or on the best method for delivering these interventions in terms of intensity (frequency of sessions) and length of intervention (number of sessions and length of time for follow up).” 2. The aims and objectives have been re-worded to offer more specific details. The protocol has been changed from: • To investigate the recruitment process including eligibility assessment, the recruitment interview and the views of children and parents about the recruitment process. • Assess the number of eligible children, the number of children approached, the number recruited and the number retained in the first 6 months of the study. • To assess issues of retention and interviewing those who drop out of the study. • Assess the acceptability (satisfaction and compliance) of graded exercise therapy and activity management. • Assess the feasibility and acceptability of using accelerometers to measure physical activity in children with CFS/ME. • Evaluate whether the two treatments are distinct and being delivered in a consistent manner across centres. to: • Assess the number of eligible children, the number of children approached, the number recruited and the number retained in the first 6 months of the study. • Identify barriers and facilitators to trial recruitment with a view to addressing barriers where possible. • Explore issues of retention and understand why people drop out of the study. • Assess the acceptability (satisfaction and adherence) of GET and activity management. • Assess the feasibility and acceptability of using accelerometers to measure physical activity in children with CFS/ME. • Evaluate whether the two interventions are distinct and being delivered in a consistent manner across centres. 3. The protocol previously stated that the recruitment discussion would be carried out by a “research nurse”. This has been amended to enable any trained member of the research team to undertake these calls, therefore the wording is more generic e.g. “member of the research team” or “the recruiter”. 4. The protocol currently states that recruitment discussion will be carried out over the phone or face-to face. We have updated the protocol to indicate that the recruitment discussion may also take place over Skype. This is because the service is routinely using Skype and potential participants and their parents have requested this. 5. The protocol previously stated that activity management would be carried out by CFS/ME specialists that are not physiotherapists. The protocol has been amended to state that any activity management trained CFS/ME specialist, including physiotherapists, may deliver this intervention. 6. The protocol previously stated that graded exercise therapy (GET) would only be carried out by GET-trained physiotherapists. The protocol has been amended to state that any CFS/ME specialist who has been trained in GET (including occupational therapists, nurses, psychologists) may deliver this intervention. 7. The protocol previously stated: In both treatment arms, children, their parents/carers and the clinician providing treatment will choose the number of follow-up sessions (between 8 and 12) and the frequency of appointments (every 2–6 weeks) within a maximum length of treatment of 1 year. The amended protocol offers estimations for frequency and duration and clarifies that trial intervention should be carried out in line with standard clinical practice: In both treatment arms, children, their parents/carers and the clinician providing treatment will choose the number of follow-up sessions (estimated to be between 8 and 12) and the frequency of appointments (every 2–6 weeks), in line with standard clinical practice. 8. We have changed the protocol to indicate that therapist may offer intervention sessions via Skype. This is because this has become routine clinical practice and patients and parents have requested this. In this instance, sessions will still be audio recorded. Children will be asked to provide consent to the audio recording of treatment session on paper forms, to be returned by post, or on-line via the redcap system. 9. The protocol previously stated: We will assume that the intervention is not acceptable for participants who DNA or have late cancellations for > 25% of follow-up appointments. On the recommendation of the trial management group, this has been changed to: We will assume that those who did not attend (or cancelled within 24 h) three or more consecutive appointments or 50% of appointments did not find the interventions acceptable. 10. The outcome measure EQ-5D-5 L has been changed to EQ-5D-Y. The EQ-5D-Y is designed and validated for use with younger populations. Both the trial management group and trial steering committee advised that the EQ-5D-Y should be used in this trial. 11. The trial management group recommended that wear diaries should only be sent to participants in the initial stages of the study to determine the validity of accelerometer data. Beyond this, the diaries would be an additional burden for participant with little added benefit. The protocol previously stated: Participants will be asked to complete a log of wear time (time worn and time taken off) which can be integrated with their activity/exercise diaries. This has been updated to: In the initial stage of the study approximately 10 participants will be asked to complete a log of wear time (time worn and time taken off). This wear diary data will be compared against accelerometer data to check for the validity/ reliability of using accelerometers with young people with CFS/ME. 12. Further details about the analysis of accelerometer data have been provided. The following have been added: The accelerometer data will be processed to identify mean minutes of sedentary, light and moderate to vigorous intensity physical activity per day using established accelerometer cut-off points and protocols16 24 . The mean accelerometer counts per minute, which provides an indication of the volume of physical activity in which the participant engages, will also be calculated using established methods. 13. We have changed the protocol to indicate that the qualitative interviews will also be offered over Skype. If the interview takes place over Skype,s children will be asked to provide consent on paper forms, to be returned by post, or on-line via the redcap system. 14. Stop-go criteria for the study has been added: Proposed stop criteria for MAGENTA: We will not proceed to the full trial if at 12 months (end of August 2016), one of the following is true: • We have recruited less than 70 children (~ 70% of the target specified) AND if the qualitative data collected suggests that we cannot improve recruitment by changing recruitment methods • The 6-month follow-up is less than 80% and AND if the qualitative data suggests we cannot improve follow-up. • The qualitative data suggests the interventions are not acceptable to children and/or their parents. • If the DSMC recommend the trial is stopped for safety reasons and if the TSC agree with this decision. 15. Further details about safety analysis have been added. The following are now detailed: Safety outcomes For safety outcomes, we will prospectively collect serious and non-serious adverse events defined as any clinical change or illness reported at clinic or postal follow-up. We will define a serious deterioration in health as: a decrease ≥ 20 in SF-36-PFS or scores of “much” or “very much worse” on the Clinical Global Impression scale; clinician reported serious deterioration in health; or withdrawal from treatment because of feeling worse. Safety outcomes will be analysed by the Data and Safety Monitoring Committee (DSMC) and reported to the Trial Steering Committee. Frequency of analyses The first interim safety analyses will be at 10 months (before the trial transitions from the feasibility to full phase). The DSMC will decide on the second safety analyses which may be at 18 months or when 50% of children have been recruited. The safety analyses will be used to ensure that neither intervention arm is having a detrimental effect. These analyses will only investigate safety outcomes and will be conducted by an independent statistician with results provided to the Data and Safety Monitoring Committee. Data and Safety Monitoring Committee (DSMC): (3 independent experts in CFS/ME, statistics and trials) will have unblinded access to the data to make recommendations to the TSC on whether there are safety reasons to stop the trial. 16. The protocol previously gave a target sample size for all three sites combined. The new protocol offers a target sample-size for each site. 17. The protocol previously stated that the feasibility study would recruit for 13 months; this has been amended to 12 months.
3.	27/07/16	1.Changes to wording in the protocol • We have updated the Background section of out protocol to incorporate further studies in the field. We have changed “no evidence” to “little evidence”, and we have now included references to Lim and Lubitz (2002), Gordon and Lubitz (2009) and Gordon et al (2010). • We have corrected a typo “on” to “of” • We have changed the wording through from “child” or “children” to know say “Child and adolescent” or “children and adolescents” or “young person” or “young people” or “participants.” This more accurately describes our study population. • We have made additional comments about the exclusion criteria section, to provide further definition and explanation. This has changed from: “Children will be excluded if they: - are too severely affected to attend hospital appointments (and require a domiciliary assessment); - are referred for CBT at their first clinical assessment; - are unable to attend follow-up appointments; “ To: “Children and adolescents will be excluded if they are severely affected. NICE defines severe CFS/ME as individuals who are unable to do activity for themselves, or carry out minimal daily tasks only, they have severe cognitive difficulties and depend on wheelchair for mobility 5; or are referred for CBT at their first clinical assessment or are unable to attend clinical sessions. Eligibility assessment will be carried out by the clinician at assessment and confirmed by the recruiting researcher.” 2. Amendments to health economics forms We have had a poor response rate to our health economic forms, and we want to address this issue during the feasibility stage. We have been working closely with our Trial Management Group and our Health Economists and have developed a second version of the baseline, 6- month and 12-month questionnaires. The new forms continue to capture our primary outcomes. However, we have removed unnecessary questions, streamlined others, changed wording and have re-structured the forms to make them more user-friendly. We have attached both the original versions and second versions of these forms. We have changed the wording in the protocol to reflect changes. From: “Both parents will be asked to complete 3 inventories on-line at baseline, 6 and 12 months follow-up including: socioeconomic status (baseline only); an adapted 4 item Work Productivity and Activity Impairment Questionnaire (General Health V2.0 [WPAI:GH])30 and an adapted existing healthcare resource use questionnaire to measure health service use (e.g. GP or specialist care), educational service (e.g. school counsellor) and travel costs.” To: “Both parents will be asked to complete 3 inventories on-line at baseline, 6 and 12 months follow-up including: an adapted 6-item Work Productivity and Activity Impairment Questionnaire (General Health V2.0 [WPAI:GH])30 and an adapted existing healthcare resource use questionnaire to measure health service use (e.g. GP or specialist care), educational service (e.g. school counsellor) and travel costs.” 3. Closing down the Cambridge site We are unable to recruit from the Cambridge site because there is no support for recruitment and the clinical team is unable to deliver the trial as per protocol. Our protocol has been amended. It now states that we will be recruiting from Bath and Newcastle only.

## Abbreviations

CBT: Cognitive behavioural therapy
CFS: Chronic fatigue syndrome
DSMC: Data Safety Monitoring Committee
GET: Graded exercise therapy
ME: Myalgic encephalomyelitis
NICE: National Institute for Health and Care Excellence
RCT: Randomised controlled trial
REDCap: Research Electronic Data Capture
TMG: Trial Management Group
TSC: Trial Steering Committee
YPAG: Young Persons Advisory Group
