# Supplementary figures and images for: Results of the feasibility phase of the managed activity graded exercise in teenagers and pre-adolescents (MAGENTA) randomised controlled trial of treatments for chronic fatigue syndrome/myalgic encephalomyelitis
Source: Pilot Feasibility Stud. 2019 Dec 19;5:151. doi: 10.1186/s40814-019-0525-3 (PMC6924066; doi:10.1186/s40814-019-0525-3)

## CONSORT: Centre Two

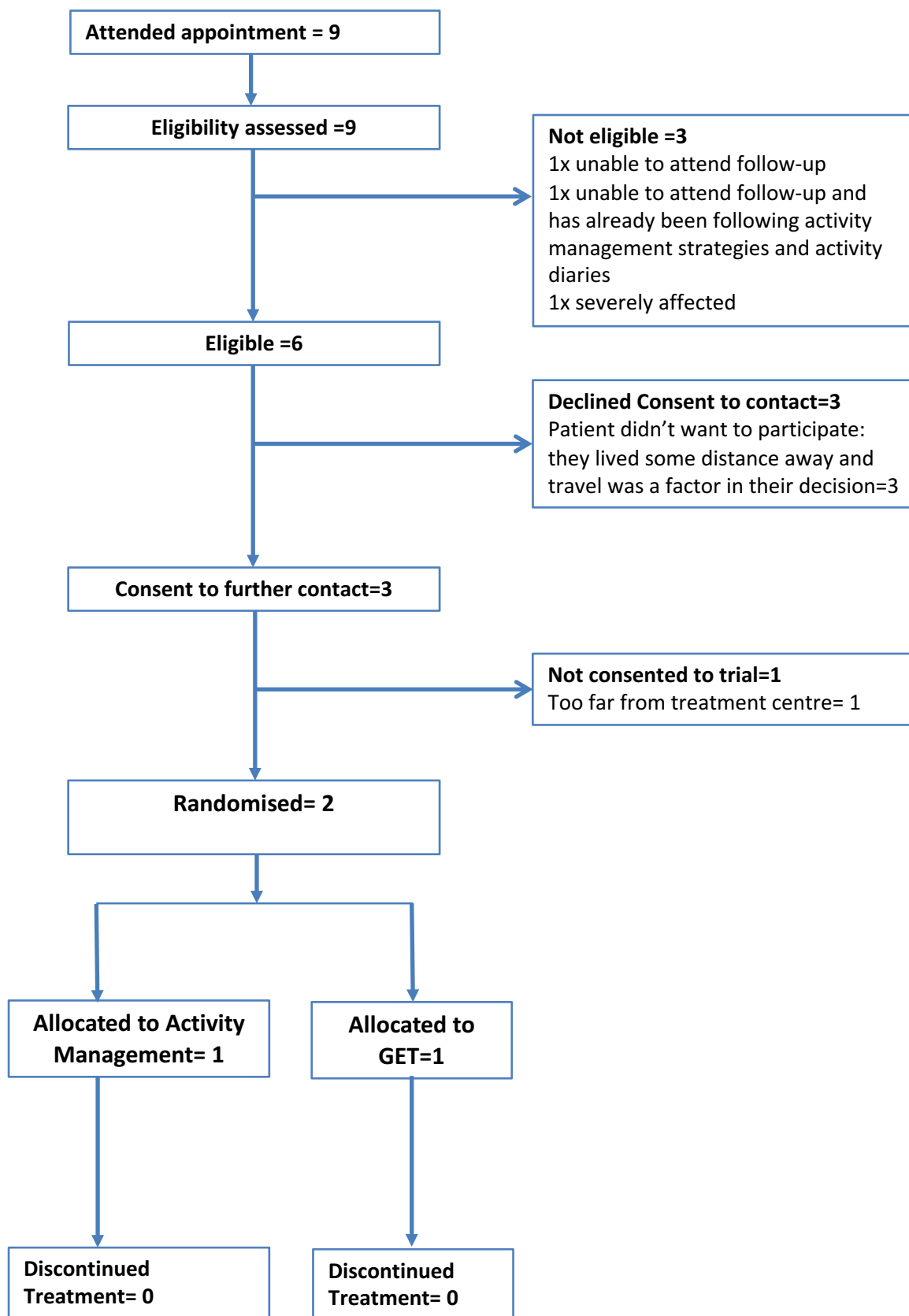

Supplement: Supplementary file 1 — Additional file 1:. CONSORT: Centre Two. [file 40814_2019_525_MOESM1_ESM.pdf]

## CONSORT: Centre Three

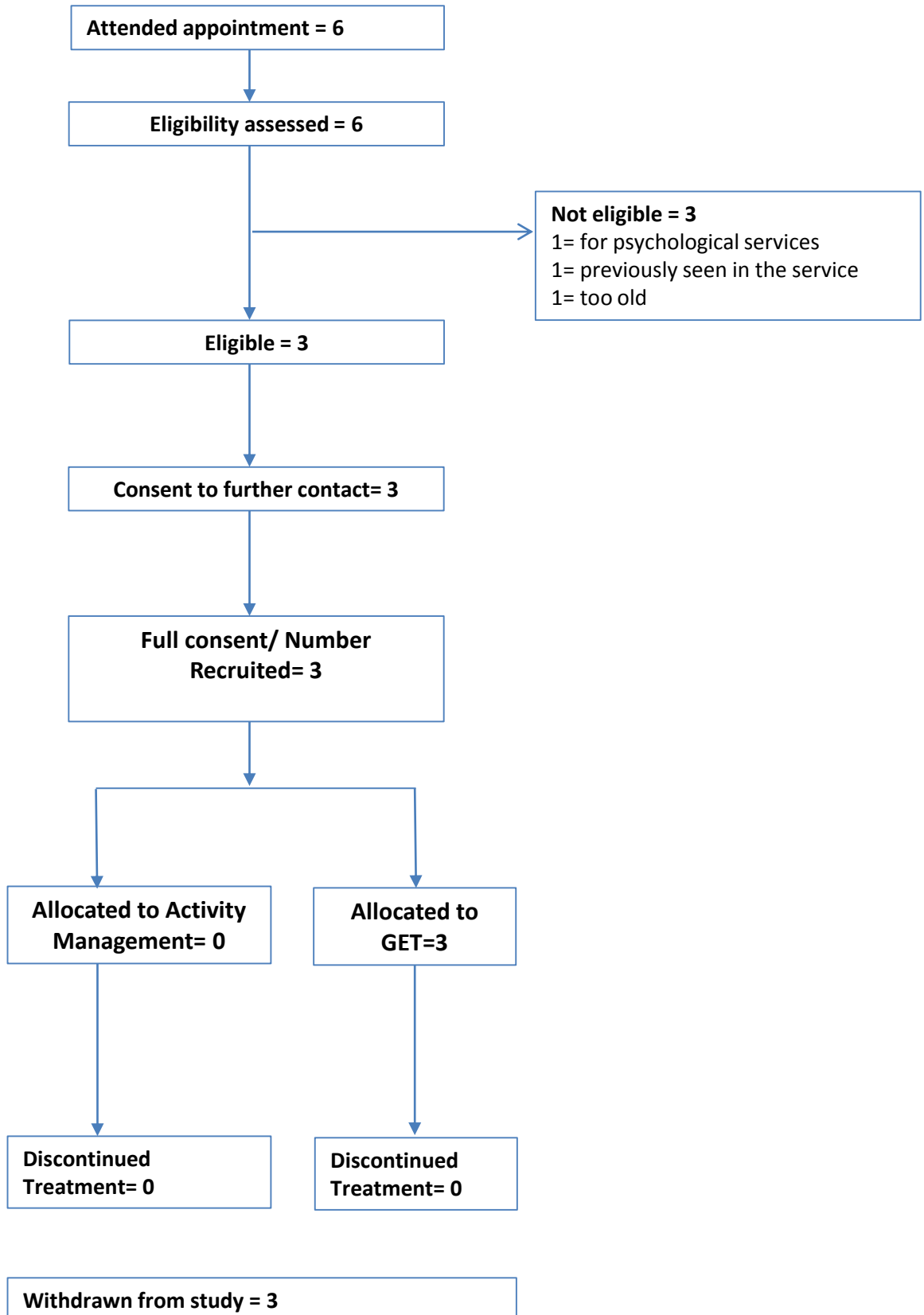

Supplement: Supplementary file 2 — Additional file 2:. CONSORT: Centre Three. [file 40814_2019_525_MOESM2_ESM.pdf]
